# Danish value sets for the EORTC QLU-C10D utility instrument

**DOI:** 10.1007/s11136-023-03569-w

**Published:** 2024-01-06

**Authors:** Jens Lehmann, Leslye Rojas-Concha, Morten Aagaard Petersen, Bernhard Holzner, Richard Norman, Madeleine T. King, Georg Kemmler, the EORTC Quality of Life Group

**Affiliations:** 1 University Hospital of Psychiatry II, Medical University of Innsbruck, Anichstraße 36, 6020 Innsbruck, Austria; 2 University Hospital of Psychiatry I, Medical University of Innsbruck, Innsbruck, Austria; 3 Palliative Care Research Unit, Department of Geriatrics and Palliative Medicine GP, Bispebjerg & Frederiksberg Hospital, University of Copenhagen, Copenhagen, Denmark; 4 School of Public Health, Curtin University, Perth, WA Australia; 5 School of Psychology, Faculty of Science, University of Sydney, Sydney, NSW Australia

**Keywords:** Cancer, Discrete choice experiment, Quality of life, Utility, Denmark, EORTC QLQ-C30

## Abstract

**Purpose:**

In this study, we developed Danish utility weights for the European Organisation for Research and Treatment of Cancer (EORTC) QLU-C10D, a cancer-specific utility instrument based on the EORTC QLQ-C30.

**Methods:**

Following a standardized methodology, 1001 adult participants from the Danish general population were quota-sampled and completed a cross-sectional web-based survey and discrete choice experiment (DCE). In the DCE, participants considered 16 choice sets constructed from the key 10 dimensions of the QLU-C10D and chose their preferred health state for each one. Utility weights were calculated using conditional logistic regression with correction for non-monotonicity.

**Results:**

The sample (*n* = 1001) was representative of the Danish general population with regard to age and gender. The domains with the largest utility decrements, i.e., the domains with the biggest impact on health utility, were physical functioning (− 0.224), pain (− 0.160), and role functioning (− 0.136). The smallest utility decrements were observed for the domains lack of appetite (− 0.024), sleep disorders (− 0.057), and fatigue (− 0.064). Non-monotonicity of severity levels was observed for the domains sleep disturbances, lack of appetite, and bowel problems. Deviations from monotonicity were not statistically significant.

**Conclusion:**

The EORTC QLU-C10D is a relatively new multi-attribute utility instrument and is a promising cancer-specific health technology assessment candidate measure. The country-specific Danish utility weights from this study can be used for cost-utility analyses in Danish patients and for comparison with other country-specific utility data.

**Supplementary Information:**

The online version contains supplementary material available at 10.1007/s11136-023-03569-w.

## Background

In healthcare systems with finite resources, cost-utility analyses (CUA) are recommended to supplement decision-making on which treatments should be prioritized and reimbursed to maximize societal healthcare benefits [5]. To evaluate the benefits of new or existing treatments in the context of CUA, quality-adjusted life years (QALYs) are used. QALYs consider not only the length of duration spent in a health state (i.e., in cancer often time until death) but also weigh-in individuals’ health-related quality of life (HRQOL). In this framework, individual health states can vary on a level between perfect health (1) and a value equal to being dead (0). Values below 0, indicating a health state perceived as worse than being dead, are possible as well in some instances [10]. In other words, such a health state would be so poor that death would be preferable over living in that state and the utility value assigned to this health state is negative [42]. The QALY metric is used in CUA and provides more comprehensive information on the actual costs and benefits of different treatments. Especially in cancer care, where incremental survival gains of new treatments can be low and treatment costs are steadily rising [37], considering patient HRQOL in CUA is crucial to a comprehensive analysis.

Preference-based measures (PBMS) are frequently used to determine the preferences different populations assign to different aspects of HRQOL. They can be generic, like the EQ-5D [12] or the Short-Form 6 Dimensions (SF-6D) [3, 6], which allows them to be used and compared across different populations and condition groups. At the same time, these PBMs may be less suitable for some cancer populations as they may be less capable to measure specific issues or aspects of diseases that may be particular to certain populations [4, 13, 27]. Disease-specific PBMs can favorably be used to capture such domains [26].

To meet the demand for disease-specific PBMs, the European Organisation for Research and Treatment of Cancer (EORTC) and the Multi-Attribute Utility in Cancer (MAUCa) Consortium have jointly developed a new, cancer-specific PBM, the EORTC Quality of Life Core 10 Dimensions (EORTC QLU-C10D) [23]. It is based on the most widely used questionnaire in cancer clinical trials [in 66% of registered trials [17]], the EORTC Quality of Life Core Questionnaire (EORTC QLQ-C30 [1]. Based on this questionnaire, the QLU-C10D covers both general functioning domains and cancer-specific symptoms.

To calculate health utilities for different populations, utility weights, which represent the health preferences of that population, are required. Utility weights can be calculated on the general population level by using preference-based methods such as standard gamble, time trade-off, and discrete choice experiments (DCEs), where DCEs are one of the most widely used methods in diverse populations [5]. As different countries and healthcare systems have different approaches to health economic evaluations and CUA [11] and health state preferences are known to differ between countries [15], country-specific utility weights should be used where available and required by agencies. For the QLU-C10D, utility weights have already been established for a number of European countries [14, 15, 18, 20, 30], Australia [24], the United States [36], Canada [29], and first Asian countries [28].

In this study, we developed utility weights for the EORTC QLU-C10D for Denmark. Historically, Danish regulators and payers consider CUA for health policy decision-making, but CUA are not mandatory for the approval or prescription of treatments [8, 38]. Notably, in 2021, the Danish Health Technology Council (Behandlingsrådet) was founded with the goal to target healthcare resources at the technologies and treatments that provide the best value for money [8]. It is expected that the evaluations by the Health Technology Council will have a major impact on the future use of technologies and healthcare devices in Denmark [8]. Regarding the assessment of HRQOL and QALYs, the Health Technology Council states that “it is not possible to give a standardized approach to how applicants can or should collect data on patients’ health-related quality of life, including which instruments are best suited for specific evaluations” [7]. However, instrument-specific validation studies in the Danish population are required for an instrument to be considered [7], which we aim to provide in the present study.

## Methods

### Study design and sample

For this study, we followed the methods for the generation of utility weights established by King et al. [24] which were also used in the valuation surveys for other countries [14, 15, 18, 20, 30]. In this cross-sectional valuation survey, participants from the Danish general population were recruited via a third-party provider online panel (https://surveyengine.com/). Participants of the survey received a small payment for their participation. We used quota sampling to obtain representativeness of the sample with regard to sex and age based on United Nations census data for Denmark [40].

All participants provided electronically signed informed consent and the study was approved by the Ethics Committee of the Medical University of Innsbruck (approval number 1079/2023).

### Survey

The survey comprised the assessment of sociodemographic data, basic clinical data (chronic diseases), the EORTC QLQ-C30, the DCE to establish Danish utility weights, the EQ-5D-5L [34], and the Kessler psychological distress scale [22]. The EQ-5D-5L and the K10 were not evaluated in this paper.

### EORTC QLQ-C30 and EORTC QLU-C10D

The EORTC QLU-C10D is a utility scoring algorithm that was developed by the MAUCa Consortium and the EORTC based on the EORTC QLQ-C30 [23]. The QLQ-C30 is a 30-item questionnaire for patients with cancer. It assesses functional health (physical functioning, role functioning, social functioning, emotional functioning, cognitive functioning), symptom burden (fatigue, pain, nausea/vomiting, sleep disturbances, dyspnea, appetite loss, constipation, diarrhea, financial impact), and global health/quality of life. For the QLU-C10D, 10 key domains from the QLQ-C30 were selected: physical functioning, role functioning, social functioning, emotional functioning, pain, fatigue, sleep disturbances, appetite loss, nausea, and bowel problems (which merges the constipation and diarrhea scales from the QLQ-C30). As in the QLQ-C30, items in the QLU-C10D have four possible levels ranging from “Not at all” to “Very much” impairment in that domain. With 10 domains with each four levels of severity, the QLU-C10D covers a total of 4^10 = 1,048,576 different health states. Supplementary Table 1 shows the classification system of the QLU-C10D. The robustness of the QLU-C10D methodology and its psychometric properties like test–retest reliability have been demonstrated in past studies [16, 31, 33].

Due to its basis on the QLQ-C30, the QLU-C10D is backward-compatible with the QLQ-C30, meaning that health utilities can be calculated from QLQ-C30 data if utility weights for the respective population are available.

### Discrete choice experiment (DCE)

Utilities were elicited by DCE in this study, which was constructed analogously to the one used by King et al. [24]**.** Out of a set of 960 possible choice sets, participants were randomly assigned to complete 16 choice sets to limit participant burden. For each choice set, participants had to choose one of two scenarios. Scenarios were set up and described based on the 10 domains from the QLU-C10D and also included survival time as an anchor. Survival time varied between 1, 2, 5, or 10 years (wording: “You live in this health state for X years, and then die”). To keep the choice sets comparable and participant burden acceptable, the scenarios of a single choice set only differed in 5 out of the 11 aspects (10 QLU-10D domains and survival time) which was previously found to be an adequate format [33]. The differing aspects were highlighted to increase their visibility for participants. See Supplementary Fig. 1 for an example of the DCE display in English language.

In the DCE section of the survey, randomization occurred at two levels: (i) each participant was assigned 16 choice sets out of the 960 available without duplication; (ii) the assignment of options as Situation A or B within each choice set was randomized to minimize potential ordering biases. It is worth noting that the presentation order of dimensions remained consistent, as prior research indicated that dimension order did not consistently impact the utility weights for the QLU-C10D [31].

### Statistical analysis

Descriptive statistics are reported for the sample characteristics (i.e., demographic information, clinical information). Chi-square tests were used to compare the sample characteristics of gender, age, and education with national census data [39]. To calculate utility weights, we followed an established procedure [2] that was also used in previous studies on the EORTC QLU-C10D, [15, 18, 33]. The utility (*U*) of an option *j* in the choice set *s* for a respondent *i* can be described by the following formula:

$$Uisj = \alpha TIMEisj + \beta X^{\prime}isjTIMEisj + \varepsilon isj$$

*TIMEisj* is the survival time in option *j* and *X’isj* is a set of dummies related to the levels of the corresponding health state. Errors (*εisj*) are assumed to be independent and identically Gumbel-distributed [2, 24]. DCE data were analyzed using a conditional logit model with cluster sandwich estimators to adjust for correlated observations within respondents. Analogous to previous valuation studies, estimates were converted into utility decrements based on the ratio of health state parameters (*β*) and the time coefficient (*α*) to reflect the trade-off between HRQOL and survival. Finally, we ran an additional analysis to correct for non-monotonic levels in the EORTC QLU-C10D utility weights (i.e., for domains where the severity levels did not show an increasing decrement in utility weight) in line with previous valuation studies.

In a recent review of DCE studies in health state valuation by Wang et al. [41], the majority of studies (48/65, 74%) used conditional logit models. In our study, we also relied on a conditional logit models because its parameterization is more suited our purpose (for public decision-making) compared to mixed logit models that focus on the distribution of individual preferences. Analyses were run using SPSS version 27.0 (IBM Corp., Armonk, NY, USA).

### Data quality checks

We recorded the total time it took respondents to complete the survey and the number of participants that only chose the A or only chose the B option in the DCE. We also computed an additional model in which we excluded the fastest 10% of participants and compared that to the model with all participants.

## Results

### Survey completion

Between 04.08.2021 and 24.08.2021, 1744 participants initiated the survey. Of those, 269 (15.4%) participants were excluded due to the quota sampling, 417 (23.9%) were excluded due to timing out (being idle mid-survey for too long and not progressing further in the survey), and 57 (3.3%) were excluded because they did not finish the survey (actively closed the survey before completion). The final sample consisted of 1001 participants.

### Socio-demographic characteristics

Socio-demographic and data on chronic diseases are shown in Table 1. For gender and age, the sample was representative of the Danish general population (both *p* > 0.975). Regarding education, the sample showed significantly higher education levels compared to the general population (*p* < 0.001) and most participants had upper secondary (47.8%) or tertiary education (41.9%). Most participants (59.9%) reported being married or in a steady relationship. About one-third (35.5%) of participants reported having at least one chronic disease. The most commonly reported diseases were arthritis or rheumatism (11.0%), respiratory diseases (10.4%), and diabetes (8.7%).

**Table 1:** Patient characteristics

	Sample (*n* = 1001)		Population^a^	Comparison		
	*n*	%	%	*χ*^2^	df	*p*
*Gender*						
Male	500	49.95	50.0	0.001	1	0.975
Female	501	50.05	50.0			
*Age group*						
18–29	202	20.2	20.6	0.198	5	0.999
30–39	157	15.7	15.8			
40–49	168	16.8	16.8			
50–59	184	18.4	18.0			
60–69	152	15.2	15.0			
70–79	138	13.8	13.8			
*Education*						
Primary, lower secondary	103	10.3	24.4	108.2	2	< 0.001
Upper secondary	479	47.8	39.8			
Tertiary (bachelor, master, PhD)	419	41.9	35.8			
*Relationship status*						
Single	267	26.7				
Married or in steady relationship	598	59.8				
Separated or divorced	94	9.4				
Widowed	41	4.1				
Any chronic diseases	345	35.5				
Asthma, emphysema, or chronic bronchitis	104	10.4				
Arthritis or rheumatism	110	11.0				
Cancer diagnosed in last 3 years	22	2.2				
Diabetes	87	8.7				
Digestive problems (such as ulcer, colitis, or gallbladder disease)	60	6.0				
Heart trouble (such as angina, congestive heart failure, or coronary artery disease)	59	5.9				
HIV illness or AIDS	0	0.0				
Kidney disease	8	0.8				
Liver problems (such as cirrhosis)	14	1.4				
Stroke	10	1.0				

^a^Population statistics for Denmark [39]

### Feedback question regarding the discrete choice experiments

Participants reported using various strategies to choose between health states. The most common strategies were focusing on the highlighted aspects which differed between the two health states (33%), thinking about most of the aspects (25%) and focusing on only a few selected aspects (15%). Among those who used a different strategy (*n* = 165), most reported via open text that they focused on the desire to live as long as possible (68%) or live with less symptom burden (13%).

### Utility decrements and calculation of QLU-C10D utilities

**Fig. 1:**
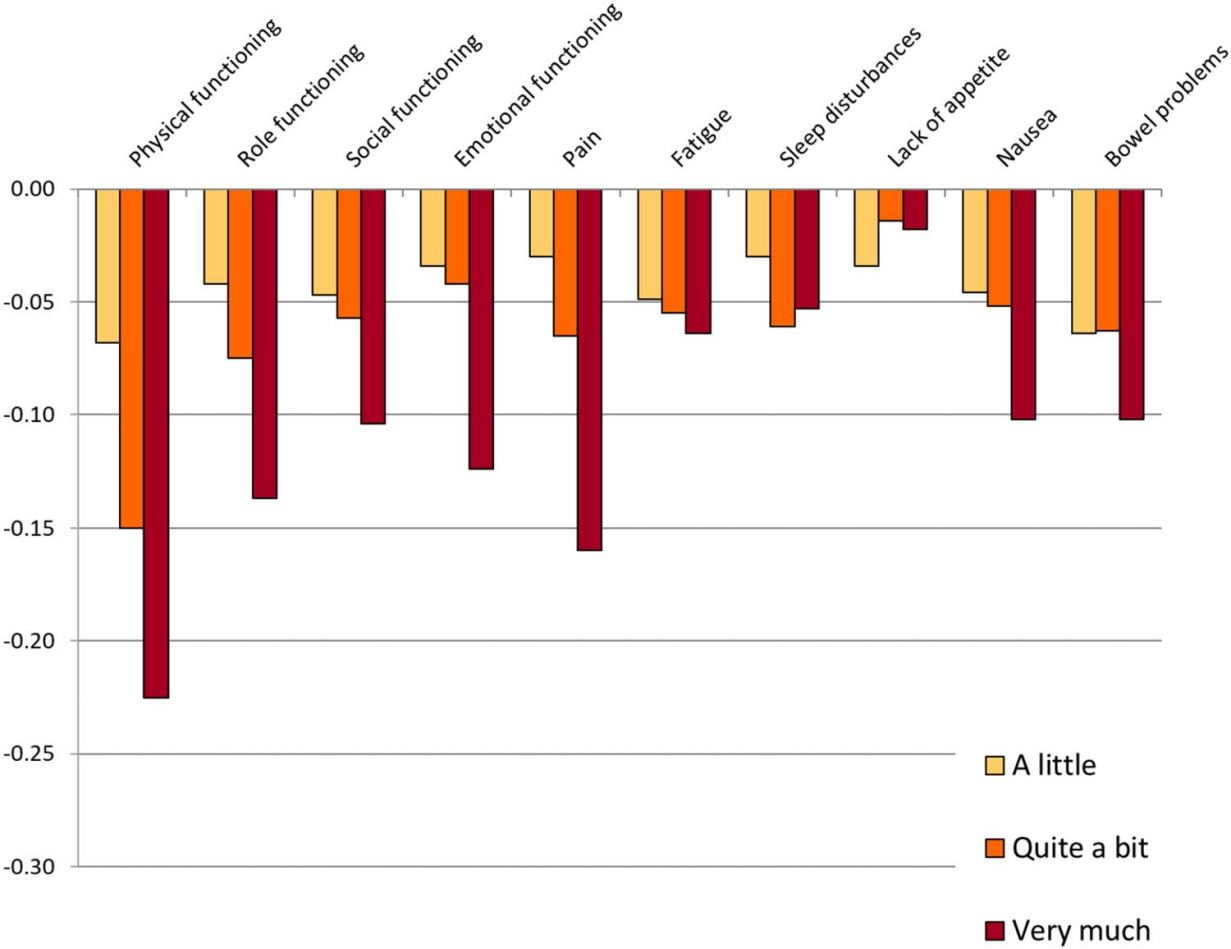
Raw decrements without adjustment for monotonicity

**Fig. 2:**
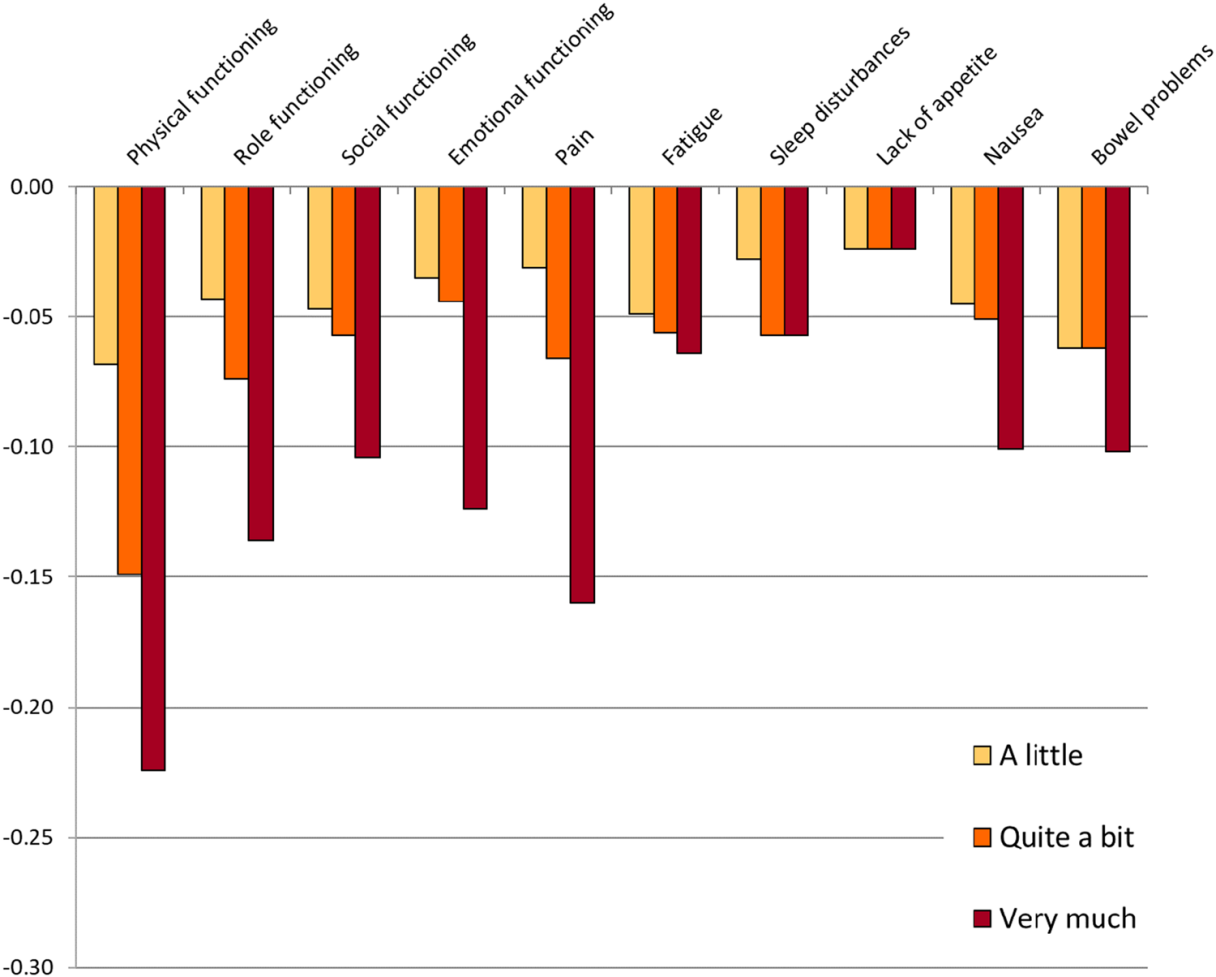
Decrements with adjustment for monotonicity of levels

The DCE analyses without correction for monotonicity are shown in Fig. 1 and Table 2. The analyses with correction for monotonicity are shown in Fig. 2 and Table 2. We report the utility decrements for each domain and severity level. For the level “not at all” (i.e., no impairment), the decrement is, by definition, 0. Actual utilities corresponding to individual health states can be calculated by subtracting the decrements from Table 2 from 1. For example, the utility for a health state where all domains would have a severity level of “quite a bit” (33333 33333), based on the utility decrements with correction for monotonicity, would be calculated as follows:

**Table 2:** Weighted DCE analysis by conditional logistic regression—uncorrected and corrected for monotonicity

Parameter	Severity level^a^	No correction for monotonicity			Corrected for monotonicity		
		Parameter estimate	SE	Utility decrement	Parameter estimate	SE	Utility decrement
Time coefficient (*α*)	(linear)	0.525	0.039		0.525	0.038	
Physical functioning	2	− 0.036**	0.014	− 0.068	− 0.036**	0.014	− 0.068
	3	− 0.079**	0.014	− 0.150	− 0.078**	0.014	− 0.149
	4	− 0.118**	0.013	− 0.225	− 0.118**	0.013	− 0.224
Role functioning	2	− 0.022*	0.011	− 0.042	− 0.022*	0.011	− 0.043
	3	− 0.039**	0.011	− 0.075	− 0.039**	0.011	− 0.074
	4	− 0.072**	0.010	− 0.137	− 0.071**	0.010	− 0.136
Social functioning	2	− 0.024*	0.010	− 0.047	− 0.025*	0.010	− 0.047
	3	− 0.030**	0.011	− 0.057	− 0.030**	0.011	− 0.057
	4	− 0.055**	0.010	− 0.104	− 0.055**	0.010	− 0.104
Emotional functioning	2	− 0.018	0.011	− 0.034	− 0.018	0.011	− 0.035
	3	− 0.022*	0.011	− 0.042	− 0.023*	0.011	− 0.044
	4	− 0.065**	0.010	− 0.124	− 0.065**	0.010	− 0.124
Pain	2	− 0.016	0.011	− 0.030	− 0.016	0.011	− 0.031
	3	− 0.034**	0.012	− 0.065	− 0.035**	0.012	− 0.066
	4	− 0.084**	0.010	− 0.160	− 0.084**	0.010	− 0.160
Fatigue	2	− 0.026	0.010	− 0.049	− 0.026	0.010	− 0.049
	3	− 0.029**	0.011	− 0.055	− 0.030**	0.011	− 0.056
	4	− 0.034**	0.009	− 0.064	− 0.034**	0.009	− 0.064
Sleep disorders	**2**	− **0.016**	**0.011**	− **0.030**	− 0.015	0.011	− 0.028
	**3**	− 0.032**	**0.011**	− **0.061**	− 0.030**	0.010	− 0.057
	**4**	− 0.028**	**0.011**	− **0.053**	− 0.030**	0.010	− 0.057
Lack of appetite	**2**	− **0.018**	**0.010**	− **0.034**	− 0.012	0.008	− 0.024
	**3**	− **0.007**	**0.011**	− **0.014**	− 0.012	0.008	− 0.024
	**4**	− **0.009**	**0.009**	− **0.018**	− 0.012	0.008	− 0.024
Nausea	2	− 0.024*	0.010	− 0.046	− 0.024*	0.010	− 0.045
	3	− 0.027*	0.011	− 0.052	− 0.027*	0.011	− 0.051
	4	− 0.053**	0.010	− 0.102	− 0.053**	0.010	− 0.101
Bowel problems	**2**	− 0.034**	**0.011**	− **0.064**	− 0.033**	0.010	− 0.062
	**3**	− 0.033**	**0.011**	− **0.063**	− 0.033**	0.010	− 0.062
	**4**	− 0.054**	**0.009**	− **0.102**	− 0.054**	0.009	− 0.102

^a^Level 2 = a little; Level 3 = quite a bit; Level 4 = very much, *SE* standard error, *p < 0.05; **p < 0.01. Levels non-conforming to monotonicity before correction are in bold

*n* = 1001 respondents

$$\begin{aligned}&1 - 0.149 - 0.074 - 0.057 - 0.044 - 0.066 - 0.056\\ & \quad - 0.057 -0.024 - 0.051 - 0.062 = 0.360\end{aligned}$$

The utility score for the worst possible health state (44444 44444), or PITS state (i.e., worst attainable health), is calculated as follows:

$$\begin{aligned}1 - 0.224 - 0.136 - 0.104 - 0.124 - 0.160 - 0.064\\ & \quad - 0.057 - 0.024 - 0.101 - 0.102 =-0.096\end{aligned}$$

The largest utility decrements for the severity level “very much” were observed for the domains physical functioning (− 0.224), pain (− 0.160), role functioning (− 0.136), and emotional functioning (− 0.124). Notably, the cancer-specific domains nausea (− 0.101) and bowel problems (− 0.102) also had decrements around − 0.1. The smallest utility decrements for the severity level “very much” were observed for the domains lack of appetite (− 0.024), sleep disorders (− 0.057), and fatigue (− 0.064). The overall rank order of domains for the other severity levels was similar.

Non-monotonicity of levels was observed for the domains sleep disorders, lack of appetite, and bowel problems. However, none of the deviations from monotonicity were statistically significant. In the model adjusted for monotonicity, the levels showing non-monotonicity were set to be equal (e.g., sleep disorders level 3 = level 4).

### Data quality checks

The mean time to complete the survey was 16.0 min with a standard deviation of 10.0 min (median 13.6, inter-quartile range 9.5–19.7 min). Excluding the fastest 10% of respondents (≤ 7.03 min) from the analysis did not change the utility decrements and results were almost identical with the analysis that included all respondents (complete data available upon request from the authors).

Few respondents (1.7% of 1001) chose either only the A or only the B option in the DCE, which is comparable to our previous studies [36].

## Discussion

In this study, we developed Danish utility weights for the EORTC QLU-C10D from a sample of the general population. We recruited a panel sample that was representative of the Danish general population in regard to age and gender. The domains with the largest utility decrements that had the biggest impact on health utility were physical functioning, pain, and role functioning.

### The EORTC QLU-C10D and relevance for Denmark

The Danish healthcare system combines universal protection and equal access to healthcare for all citizens, providing high-quality services free of charge. It promotes, among other things, preventive examinations for some diseases, especially to detect cancer in early stages and provide better treatments to patients. The total socioeconomic cost of Danish cancer patients is approximately DKK 12 billion (or roughly € 1.6 billion) over a 5-year period, including direct healthcare costs, and lost income for patients and potential partners [25]. These costs are expected to increase due to the increased incidence of cancer, and therefore, the EORTC QLU-C10D may facilitate cost-utility analysis of cancer interventions in different healthcare settings. Further instructions on how to use the EORTC QLU-C10D can be found in the EORTC user manual by Gamper et al. (https://qol.eortc.org/manual/eortc-qlu-c10d-manual/).

The EORTC QLU-C10D is a relatively new multi-attribute utility instrument (MAUI) and is a promising candidate measure that is cancer-specific. Compared to generic MAUIs like the EQ-5D which offer the advantage of making comparisons between certain condition groups, specific MAUIs might more effectively capture aspects that are particular to a certain condition or disease. A 2020 review of health utility instruments that are used and recommended by national health technology assessment (HTA) guidelines found that generic MAUIs (meaning not focused on a specific disease) like the EQ-5D are still preferred in half of the reviewed guidelines [21]. However, the review also found that about half of the national guidelines do not express a preference for a specific MAUI but do recommend calculating utilities using national preference weights. This highlights the necessity for national utility weights for different countries. In this context, two Danish value sets are available for the EQ-5D-3L [43] and the EQ-5D-5L [19] but so far, no dataset was available for the EORTC QLU-C10D. One considerable advantage of the EORTC QLU-C10D is that it can be used in any cancer trial that assesses the EORTC QLQ-C30, which is the most commonly used quality of life questionnaire in published and registered clinical trials by a large margin [17]. In other words, the EORTC QLU-C10D health utilities can be calculated using patients' EORTC QLQ-C30 scores, even retrospectively for trials that have already been closed. We strongly encourage future analyses of trials using EORTC QLQ-C30 data based on the growing body of utility weights available for the EORTC QLU-C10D [15, 18, 20, 24, 30, 32, 36].

As part of the ongoing efforts to provide utility weights for the EORTC QLU-C10D, we calculated national utility weights for Denmark. The Danish Medicines Council guidelines for methods to assess new pharmaceutical states that QALYs should be used to summarize findings from health economic analyses [9] based on national weights. Sensitivity analyses are recommended if both a generic MAUI (like the EQ-5D) and disease-specific MAUI like the EORTC QLU-C10D are available [9]. With the foundation of the Danish Health Technology Council in 2021 [7], we expect a growing importance of HTA in Denmark in the near future.

### Danish utility weights and comparison to other countries

Generally, utility decrements for the EORTC QLU-C10D and the rank order of domains were similar to other European countries. Several of the findings merit in-depth discussion. In our sample, the utility decrement for physical functioning was the largest, which was also the domain with the largest decrement for many other countries; the decrement was comparable to Austria [15], Australia [24], and smaller when compared to Italy and Poland [15]. Following physical functioning, the domain with the second largest decrement was pain, which is a pattern that was, for example, also observed in, for example, Austria [15], Germany [20], Spain [14], France [30], and the United Kingdom [32].

Compared to many other countries however [14, 15], the domain with the third largest decrement in the Danish utility weights was not role functioning but emotional functioning. In general, the decrement for emotional functioning was large with − 0.124, indicating that emotional outcomes are more highly valued or considered to have a strong impact on health in the Danish population. In international comparison, only France [30], the United Kingdom [32], and Australia [24] show larger decrements for emotional functioning. Most other countries have a lower decrement for this domain [15, 18, 20, 36]. This might also be influenced by the translation of the domain, which was “Du føler dig deprimeret” and is a strong wording considered a paronym for depression in a more clinical or diagnostic sense compared to “feeling down.” However, similar wordings were also used in other countries that did not show large decrements for this domain, like the USA (“You feel depressed”) [36] or Italy (“Soffre di depressione”) [15]. Social functioning was also valued similarly high as emotional functioning at − 0.104 for the highest level, with the decrements for the lower two levels being slightly larger than the corresponding levels for emotional functioning.

Some of the domains that are considered cancer-specific like bowel problems (diarrhea, constipation) and nausea had decrements around − 0.1. These are larger than in other countries like Germany [20], Austria, Italy, and Poland [15] and similar to the Netherlands [18] and Australia [24]. Three other domains fatigue, lack of appetite, and sleep disturbances were associated with smaller utility decrements. This was also observed in other countries [15, 18, 20]. One potential explanation is that, while people from the general population do experience occasional fatigue-like symptoms or lack of appetite or sleep disturbances, they cannot relate to severe, cancer or therapy-induced fatigue or lack of appetite [36]. It is possible that patients with cancer who have actually experienced those symptoms as a consequence of antineoplastic therapy would assign a different weight to these domains. Evaluations to derive comparable utility weights from a patient population are currently ongoing.

The worst possible health state (PITS state) with the highest level of impairments in all domains had a negative value of − 0.096, indicating that it was perceived as worse than being dead in our population. This is similar to the value of the PITS state in Australia (− 0.096) [24] and the United Kingdom (− 0.083) [32].

### Strengths and limitations

One strength of our study is the large sample that was representative of the Danish general population with regard to age and gender. Moreover, we followed an established methodology to calculate the utility weights [23]. This enables cross-country comparisons of utility weights as preferences have been shown to differ between countries [35]. One limitation of our study is that the sample had a higher proportion of highly educated people compared to the general population which was also found in the evaluations of other countries [20, 36] and it is unknown if different health preferences exist in different subpopulations (e.g., in groups with different education levels). Another limitation is that we do not have sociodemographic information on respondents that did not complete the survey and who were excluded from the analysis as those respondents mostly did not complete the questions on sociodemographic information.

Non-monotonicity was found for some of the domains with smaller weights. However, none of these deviations from monotonicity were statistically significant and all could be corrected in the analysis. Finally, another possible limitation of this study is the potential cognitive burden of participants due to the length of the questionnaires included in the survey, since about a third of those who began the survey did not complete it and were excluded from the analysis.

## Conclusion

In this study, we successfully developed Danish general population utility weights that can be used for CUA. Our study adds to a growing body of literature on the EORTC QLU-C10D, a cancer-specific utility instrument based on the EORTC QLQ-C30.

### Supplementary Information

Below is the link to the electronic supplementary material.

Supplementary file1 (DOCX 351 KB)

## Data Availability

Data for this study are available upon reasonable request from the EORTC Headquarters. Available at: https://www.eortc.be/services/forms/erp/request.aspx.
