# Supplementary material for: Danish value sets for the EORTC QLU-C10D utility instrument
Source: Qual Life Res. 2024 Jan 6;33(3):831–41. doi: 10.1007/s11136-023-03569-w (PMC10894119; doi:10.1007/s11136-023-03569-w)

# Supplementary Materials

## Supplementary Table 1: Health state classification system of the QLU-C10D

| **Dimension** | **Level** | **Stem** | **Descriptor** | **QLQ-C30 item scores** |
| --- | --- | --- | --- | --- |
| Physical functioning | 1 | You have… | No trouble taking a long walk outside of the house | Item 2 (long walk) = 1 |
|  | 2 |  | No trouble taking a short walk outside of the house, but at least a little trouble taking a long walk | Item 3 (short walk) = 1 AND Item 2 ≥ 2 |
|  | 3 |  | A little trouble taking a short walk outside of the house | Item 3=2 |
|  | 4 |  | Quite a bit or very much trouble taking a short walk outside the house | Item 3 ≥ 3 |
| Role functioning | 1 | You are limited in pursuing your work or other daily activities… | Not at all | Item 6 = 1 |
|  | 2 |  | A little | Item 6 = 2 |
|  | 3 |  | Quite a bit | Item 6 = 3 |
|  | 4 |  | Very much | Item 6 = 4 |
| Social functioning | 1 | Your physical condition or medical treatment interferes with your social or family life… | Not at all | Items 26 AND 27 = 1 |
|  | 2 |  | A little | max(item26, item 27) = 2 |
|  | 3 |  | Quite a bit | max(item26, item 27) = 3 |
|  | 4 |  | Very much | max(item26, item 27) = 4 |
| Emotional functioning | 1 | You feel depressed… | Not at all | Item 24 = 1 |
|  | 2 |  | A little | Item 24 = 2 |
|  | 3 |  | Quite a bit | Item 24 = 3 |
|  | 4 |  | Very much | Item 24 = 4 |
| Pain | 1 | You have pain… | Not at all | Item 9 = 1 |
|  | 2 |  | A little | Item 9 = 2 |
|  | 3 |  | Quite a bit | Item 9 = 3 |
|  | 4 |  | Very much | Item 9 = 4 |
| Fatigue | 1 | You feel tired… | Not at all | Item 18 = 1 |
|  | 2 |  | A little | Item 18 = 2 |
|  | 3 |  | Quite a bit | Item 18 = 3 |
|  | 4 |  | Very much | Item 18 = 4 |
| Sleep | 1 | You have trouble sleeping… | Not at all | Item 11 = 1 |
|  | 2 |  | A little | Item 11 = 2 |
|  | 3 |  | Quite a bit | Item 11 = 3 |
|  | 4 |  | Very much | Item 11 = 4 |
| Appetite | 1 | You lack appetite… | Not at all | Item 13 = 1 |
|  | 2 |  | A little | Item 13 = 2 |
|  | 3 |  | Quite a bit | Item 13 = 3 |
|  | 4 |  | Very much | Item 13 = 4 |
| Nausea | 1 | You feel nauseated… | Not at all | Item 14 = 1 |
|  | 2 |  | A little | Item 14 = 2 |
|  | 3 |  | Quite a bit | Item 14 = 3 |
|  | 4 |  | Very much | Item 14 = 4 |
| Bowel problems | 1 | You… | do not have constipation or diarrhoea at all | max(item16, item 17) = 1 |
|  | 2 |  | have a little constipation or diarrhoea | max(item16, item 17) = 2 |
|  | 3 |  | have constipation or diarrhoea quite a bit | max(item16, item 17) = 3 |
|  | 4 |  | have constipation or diarrhoea very much | max(item16, item 17) = 4 |
| Duration | 1 | You will live in this health state for… | 1 year, and then die | Not applicable |
|  | 2 |  | 2 years, and then die | Not applicable |
|  | 3 |  | 5 years, and then die | Not applicable |
|  | 4 |  | 10 years, and then die | Not applicable |

**Supplementary Figure 1:** English language example screenshot from the DCE survey.


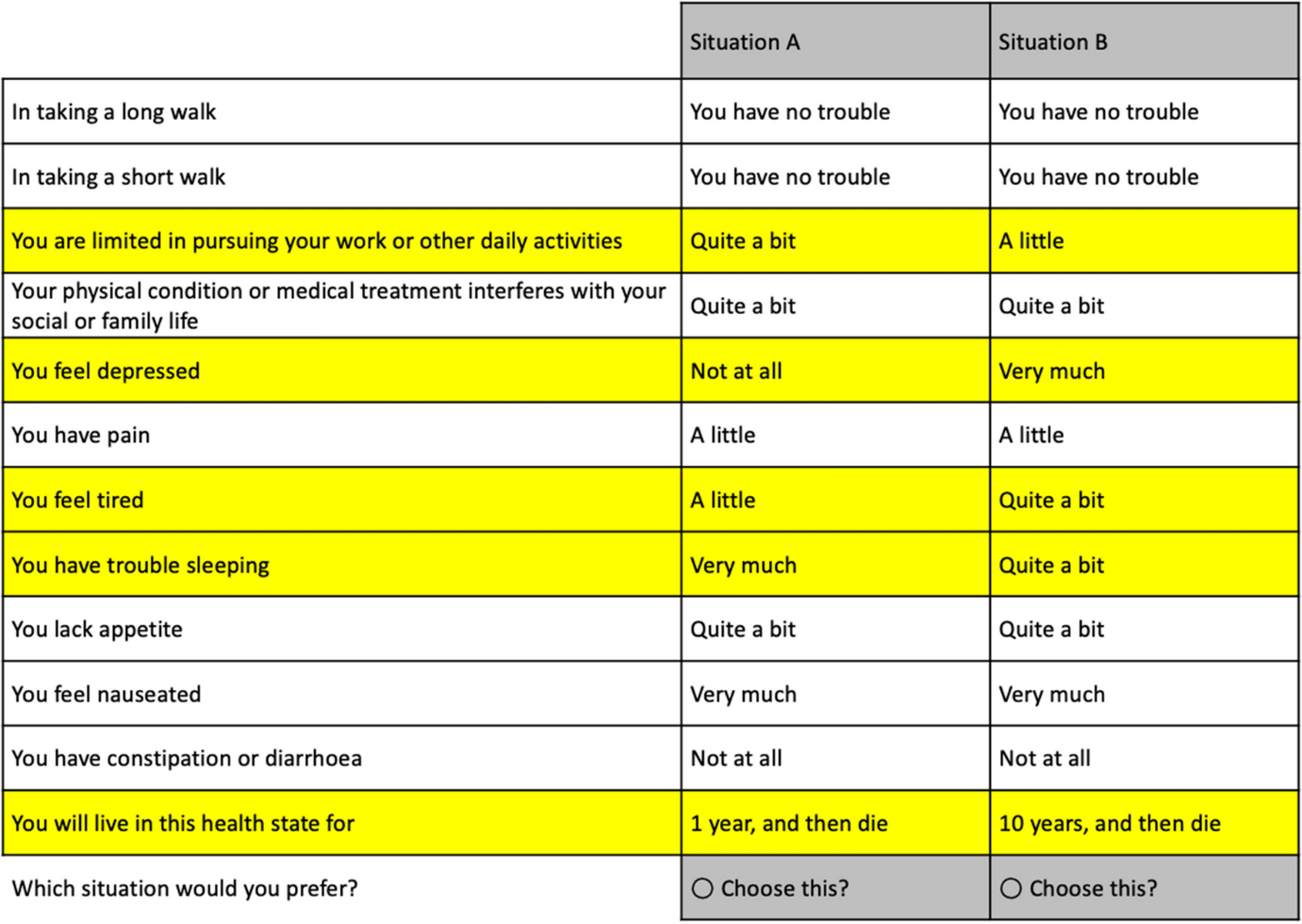

Supplement: Supplementary file 1 — Supplementary file1 (DOCX 351 KB) [file 11136_2023_3569_MOESM1_ESM.docx]
